# Interim Analysis of a Phase 2 Open‐Label Trial Assessing Burosumab Efficacy and Safety in Patients With Tumor‐Induced Osteomalacia

**DOI:** 10.1002/jbmr.4184

**Published:** 2020-11-04

**Authors:** Yasuo Imanishi, Nobuaki Ito, Yumie Rhee, Yasuhiro Takeuchi, Chan Soo Shin, Yutaka Takahashi, Hiroki Onuma, Masahiro Kojima, Masanori Kanematsu, Hironori Kanda, Yoshiki Seino, Seiji Fukumoto

**Affiliations:** ^1^ Department of Metabolism, Endocrinology, and Molecular Medicine Osaka City University Graduate School of Medicine Osaka Japan; ^2^ Division of Nephrology and Endocrinology The University of Tokyo Hospital Tokyo Japan; ^3^ Department of Internal Medicine, Severance Hospital, Endocrine Research Institute Yonsei University College of Medicine Seoul Republic of Korea; ^4^ Endocrine Center Toranomon Hospital Tokyo Japan; ^5^ Okinaka Memorial Institute for Medical Research Tokyo Japan; ^6^ Department of Internal Medicine Seoul National University Hospital Seoul Republic of Korea; ^7^ Division of Diabetes and Endocrinology Kobe University Hospital Kobe Japan; ^8^ Kyowa Kirin Co., Ltd. Tokyo Japan; ^9^ Department of Pediatrics Osaka Hospital, Japan Community Healthcare Organization (JCHO) Osaka Japan; ^10^ Fujii Memorial Institute of Medical Sciences, Institute of Advanced Medical Sciences Tokushima University Tokushima Japan

**Keywords:** CLINICAL TRIALS, OSTEOMALACIA AND RICKETS, PTH/VIT D/FGF23

## Abstract

Patients with tumor‐induced osteomalacia (TIO), an acquired paraneoplastic condition characterized by osteomalacia due to hypophosphatemia, exhibit a similar clinical picture to those with X‐linked hypophosphatemic rickets/osteomalacia (XLH). The human monoclonal anti‐fibroblast growth factor 23 (FGF23) antibody burosumab (KRN23) increases serum phosphate and improves bone turnover, fracture healing, pain, and physical function in XLH patients by inhibiting circulating FGF23; thus, burosumab is expected to be an effective treatment for TIO. We report here an interim analysis of a multicenter, open‐label, intraindividual dose‐adjustment study of burosumab (0.3 to 2.0 mg/kg every 4 weeks) in Japanese and Korean TIO patients. The primary endpoint was the fasting serum phosphate level at each visit. Key secondary endpoints were changes over time in bone biomarkers, pharmacodynamic markers, bone histomorphometric parameters, motor function, and patient‐reported outcomes. Safety was assessed based on treatment‐emergent adverse events (TEAEs). Thirteen patients received burosumab treatment, of whom 4 underwent bone biopsy. The mean dose after week 112 was approximately 1.0 mg/kg. After the first burosumab administration, mean serum phosphate levels increased and remained above the lower limit of normal and in the normal range from weeks 14 to 112. Bone biomarkers initially increased, reaching maximum values at week 16 or 24, and then gradually decreased. After burosumab treatment, patients were able to walk further (evaluated by the 6‐minute walk test), reported decreased pain levels, and showed a tendency toward healing of baseline fractures and pseudofractures. Two patients discontinued, one each due to disease progression and consent withdrawal. Burosumab was generally well tolerated, with no treatment‐related TEAEs of grade ≥3 and no treatment‐related serious AEs. In conclusion, the interim results of this first study of burosumab to treat TIO patients indicate that this drug has the potential to provide clinical benefit for patients with unresectable tumors. The full study results are eagerly anticipated. © 2020 The Authors. *Journal of Bone and Mineral Research* published by Wiley Periodicals LLC on behalf of American Society for Bone and Mineral Research (ASBMR)..

## Introduction

Tumor‐induced osteomalacia (TIO) is a rare form of fibroblast growth factor 23 (FGF23)‐related hypophosphatemic disease, in which overproduction of FGF23 leads to renal phosphate wasting and a reduction in intestinal phosphate absorption due to inappropriately suppressed 1,25‐dihydroxyvitamin D (1,25(OH)_2_D) levels, followed by hypophosphatemia.^(^ ^1^, ^2^, ^3^, ^4^ ^)^ Patients with TIO present similar clinical and biochemical features as those with other FGF23‐related hypophosphatemic diseases, such as X‐linked hypophosphatemic rickets/osteomalacia (XLH).^(^ ^5^, ^6^ ^)^ Clinical features of TIO generally include bone pain, muscle weakness, and progressive fatigue, and patients often present with fractures of the long bones, vertebrae, and ribs.^(^ ^4^, ^7^ ^)^ Bone histologic analysis and quantitative backscattered electron imaging analysis reveal marked alterations in mineralization kinetics, as evidenced by a reduced degree of mineralization and increased osteoid abundance.^(^ ^8^, ^9^ ^)^

Unlike the genetic disease XLH, TIO is an acquired paraneoplastic condition in which the tumor produces excessive levels of FGF23,^(^ ^2^, ^4^, ^7^ ^)^ and the time between onset of symptoms to diagnosis can be lengthy, taking up to 20 years.^(^ ^10^ ^)^ The causative tumors in TIO are typically benign, characterized by small size and slow growth and can occur at various sites;^(^ ^11^ ^)^ they may be difficult to identify and diagnose with standard imaging techniques.^(^ ^7^, ^11^ ^)^ For tumor lesions that can be identified and are accessible, surgical complete resection is the first treatment choice.^(^ ^2^, ^4^ ^)^ After complete resection, blood FGF23 levels decrease markedly and immediately, and biochemical parameters commonly resolve within days or weeks.^(^ ^12^ ^)^ If the tumor cannot be identified or resected, patients are treated with oral inorganic phosphate and/or active vitamin D preparations.^(^ ^4^, ^7^ ^)^ However, the efficacy of these treatments can be suboptimal, and there are safety concerns associated with them, including renal insufficiency and secondary hyperparathyroidism.^(^ ^4^, ^7^ ^)^

Burosumab (KRN23) is a fully human monoclonal antibody targeted against FGF23.^(^ ^13^ ^)^ Burosumab has shown efficacy in the treatment of XLH patients, increasing serum phosphate^(^ ^14^ ^)^ and improving bone histomorphometric measures and fracture healing.^(^ ^15^, ^16^ ^)^ Moreover, 24 weeks of burosumab treatment was associated with improvements in pain, physical function, and stiffness compared with placebo‐treated patients with XLH.^(^ ^15^ ^)^ Burosumab was approved in the United States, Canada, and the European Union for the treatment of XLH in 2018^(^ ^13^, ^17^, ^18^ ^)^ and in Japan for FGF23‐related hypophosphatemic rickets and osteomalacia in 2019.^(^ ^19^ ^)^

Burosumab is expected to be an effective treatment for TIO, particularly in patients whose causative tumors are difficult to resect surgically. Although a phase 2 clinical study (NCT02304367) in patients with TIO is ongoing in the United States,^(^ ^20^ ^)^ and a single case that suggests burosumab could improve pain and mobility was reported,^(^ ^21^ ^)^ little is known about the effect of burosumab on patients with TIO. We conducted a phase 2 open‐label trial, which is the first clinical study in TIO patients to evaluate the efficacy and safety of burosumab in this patient population.

## Materials and Methods

### Study design

**Fig 1:**
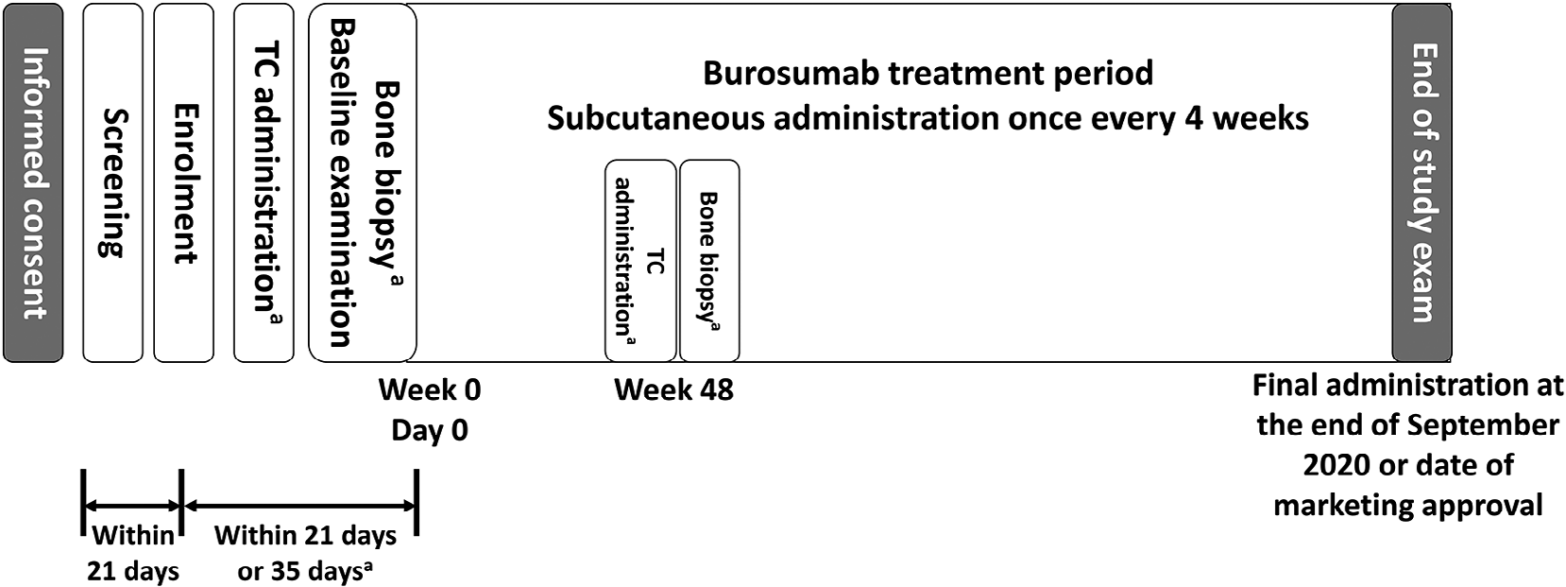
Study design. ^a^Only in patients who gave consent for bone biopsy. TC = tetracycline hydrochloride.

This is an ongoing, multicenter, open‐label, intraindividual dose adjustment study, which started in May 2016 and is planned to continue until September 2020 (Fig. 1); this report contains interim analysis data from week 112. After obtaining informed consent, potential participants underwent screening for eligibility. For eligible patients, a baseline examination was conducted, with or without bone biopsy of the ileum. Burosumab treatment was initiated 1 to 3 days after the baseline examination.

This study was an unblinded, single‐arm study. It was conducted in accordance with the principles described in the Declaration of Helsinki, all applicable local and national laws, and current Good Clinical Practice guidelines for Japan and the Republic of Korea. The protocol and all study documentation were reviewed and approved by the Institutional Review Board or Independent Ethics Committee at each investigative site (University of Tokyo Hospital, Toranomon Hospital, Osaka City University Hospital, Kobe University Hospital, Severance Hospital, and Seoul National University Hospital). All patients (and their legal representative, if patients were aged <20 years) provided written informed consent before the conduct of any study procedures. This study was registered at www.ClinicalTrials.gov under the identifier number NCT02722798.

### Patients

Key inclusion criteria were age ≥18 years; a diagnosis of TIO (elevated FGF23) with unidentified or unresectable tumor; at screening, a serum phosphate level <2.5 mg/dL (the lower limit of the normal range [LLN]), serum FGF23 level ≥100 pg/mL by Kainos assay, and renal tubular maximum reabsorption of phosphate (TmP)/glomerular filtration rate (GFR) <2.5 mg/dL; an estimated GFR (eGFR; calculated using the 3‐variable Japanese equation^(^ ^22^ ^)^) at screening ≥60 mL/min/1.73 m^2^, or ≥30 and <60 mL/min/1.73 m^2^ with no evidence of renal failure related to nephrocalcinosis; and a corrected serum calcium level at screening <10.8 mg/dL. Patients with epidermal nevus syndrome (ENS), another FGF23‐associated phosphate wasting disorder,^(^ ^23^ ^)^ were also eligible for study participation; however, no patients with ENS were enrolled.

Key exclusion criteria were use of TIO treatment (pharmacologic vitamin D metabolites or analogs, oral phosphate, aluminum hydroxide antacids, acetazolamide, or thiazide diuretics) within 14 days before screening; use of medication to suppress parathyroid hormone (eg, cinacalcet hydrochloride) within 60 days before screening; use of therapeutic monoclonal antibodies or any investigational product within 90 days before screening; pregnancy, lactation, or unwillingness to use appropriate contraception; or any medical condition that could confound the study results or endanger the participant, or anyone considered unsuitable for participation by the investigator.

### Study treatments

Burosumab was administered subcutaneously once every 4 weeks at an initial dose of 0.3 mg/kg. The dose increment from the preceding administration was set at 0.2 mg/kg and the maximum dose at 2.0 mg/kg. The dose of burosumab was adjusted at weeks 4, 8, 12, and 16 based on the serum phosphate level obtained 2 weeks before (ie, at weeks 2, 6, 10, and 14, respectively). At and after week 20, the dose of burosumab was set at the same level as at week 16. If there were concerns about safety or suboptimal efficacy, the dose could be adjusted at scheduled study visits (weeks 20, 24, 28, 32, 36, 40, 44, and at, and after, week 48). At, and after, week 92, burosumab was administered at a dosage based on current body weight and dose. Bone biopsy was performed at baseline and at week 48. In the patients who underwent bone biopsy, tetracycline hydrochloride (TC) was administered at weeks −3, −1, 45, and 47.

### Endpoints

The primary endpoint was the serum phosphate level at each time point. Blood was collected after a fast of ≥8 hours and before any treatment administration; collection times are described in the Supplemental Text (study protocol).

Secondary endpoints included the proportions of patients who achieved mean peak and trough serum phosphate values exceeding the LLN (2.5 mg/dL [0.81 mmol/L]); the proportion of patients with serum phosphate values within the normal range every 24 weeks; percentage changes from baseline in the following bone parameters at week 48: osteoid thickness, osteoid surface/bone surface ratio, osteoid volume/bone volume ratio, and mineralization lag time; changes from baseline over time in the following pharmacodynamic markers: urinary phosphate, serum 1,25(OH)_2_D, TmP/GFR, alkaline phosphatase, and markers of calcium homeostasis (serum calcium, 24‐hour urine calcium, and intact parathyroid hormone); changes from baseline over time in the following serum metabolic bone biomarkers: type I collagen C‐telopeptides (CTX), procollagen 1 N‐terminal propeptide (P1NP), bone‐specific alkaline phosphatase (BALP), and osteocalcin (OC); changes from baseline over time in motor function (6‐minute walk test [6MWT]); and changes from baseline over time in patient‐reported outcomes (brief pain inventory [BPI]). The time points for each measurement are described in the Supplemental Text (study protocol).

Exploratory endpoints were whole‐body bone scintigraphy, dual‐energy X‐ray absorptiometry scan (DXA), and standard X‐ray radiography (lateral spine, anteroposterior chest, upper arm, lower arm, hand, bilateral upper leg, bilateral lower leg, foot, and other). The time points for each measurement are described in the Supplemental Text (study protocol). Fractures and pseudofractures were evaluated by an independent reader.

Safety was assessed based on treatment‐emergent adverse events (TEAEs), coded using the Medical Dictionary of Regulatory Activities (v21.0) and graded using the Common Terminology Criteria for Adverse Events (v4.0); clinical laboratory evaluations; renal ultrasound; and echocardiogram. Immunogenicity of treatment, based on the development of anti‐burosumab antibodies, was also assessed.

### Statistical methods

For the target number of patients, based on the small numbers of TIO patients estimated in Japan^(^ ^24^ ^)^ and the Republic of Korea and the feasibility of the study (eg, frequency of study visits), the present study proposed to enroll a minimum of 6 patients. The safety analysis set comprised all patients who received a dose of burosumab; the efficacy analysis set comprised all patients who received a dose of burosumab and had at least 1 post‐baseline serum phosphate evaluation.

For the primary endpoint, descriptive statistics of serum phosphate concentration at each test time point were calculated. For the secondary endpoints, the numbers and percentages of patients who achieved the mid‐cycle and end‐cycle mean serum phosphate value exceeding the LLN were calculated; descriptive statistics of changes and percentage changes in serum phosphate from baseline were also calculated. Descriptive statistics were calculated for all other secondary outcomes. Categorical data were summarized using frequencies and percentages; continuous data included number of patients, mean, standard deviation (SD), median, minimum, and maximum. For exploratory objectives, baseline observations and changes from baseline over time were summarized. No statistical hypothesis testing was performed during this study. No imputations were made for missing data and no adjustments were made for multiplicity. Further details of the statistical methods are included in the Supplemental Text (study protocol).

## Results

### Patients

Regarding the patient disposition, 14 patients with TIO were enrolled in the study; all 14 patients provided consent for treatment, but only 4 provided consent for bone biopsy. One patient withdrew consent before treatment; therefore, 13 (92.9%) received burosumab treatment and were included in the efficacy and safety analysis sets. At the cut‐off date, 1 patient had discontinued treatment because of disease progression, 1 had withdrawn consent, and 11 (78.6%) had completed week 112 assessments.

Baseline characteristics are reported in Table 1. Of the 13 patients evaluated, 9 (69.2%) were Japanese, 7 (53.8%) were female, the mean age was 60.5 years, and the mean duration of disease was 10.0 years. All patients (100%) had previously used active vitamin D_3_, 12 (92.3%) had used inorganic phosphate, 2 (15.4%) each had used calcimimetics or had radiation treatment, and 1 (7.7%) had used octreotide. Seven patients (53.8%) had previously undergone surgery for their disease. No patients had undergone parathyroidectomy for secondary hyperparathyroidism. Tumors were identified in 7 (53.8%) patients at baseline.

**Table 1:** Patient Demographics and Clinical Characteristics at Baseline

	Safety and efficacy analysis set *N* = 13
Country	
Japan	9 (69.2)
Republic of Korea	4 (30.8)
Sex	
Female	7 (53.8)
Age (years)	
Mean (SD)	60.5 (10.8)
≥65	5 (38.5)
Height (cm)	
Mean (SD)	151.45 (10.43)
Weight (kg)	
Mean (SD)	61.38 (13.02)
Duration of disease, years (*n* = 12)	
Mean (SD)	10.0 (5.0)
Treatment for underlying disease	
Inorganic phosphate	12 (92.3)
Active vitamin D_3_	13 (100.0)
Calcimimetics	2 (15.4)
Octreotide	1 (7.7)
Radiation	2 (15.4)
Chemotherapy	0
Other	0
History of surgery	
Yes	7 (53.8)
Tumor identified at baseline	
Yes	7 (53.8)
Serum phosphate^a^ (mg/dL)	
Mean (SD)	1.62 (0.49)
Corrected serum calcium^b^ (mg/dL)	
Mean (SD)	9.02 (0.33)
1,25‐dihydroxyvitamin D^c^ (pg/mL)	
Mean (SD)	22.58 (11.87)
25‐hydroxyvitamin D^d^ (ng/mL)	
Mean (SD)	26.8 (13.2)
Parathyroid hormone,^e^ intact (pg/mL)	
Mean (SD)	112.5 (45.4)
Fibroblast growth factor 23,^f^ intact (pg/mL)	
Mean (SD)	1018.8 (1668.8)
TmP/GFR,^g^ 2‐hr urinalysis, (mg/dL)	
Mean (SD)	1.1478 (0.4259)
Alkaline phosphatase^h^ (U/L)	
Mean (SD)	424.7 (184.8)

Data are shown as *n* (%) unless otherwise stated. LLN = lower limit of normal; SD = standard deviation; TIO = tumor‐induced osteomalacia; TmP/GFR = renal tubular maximum phosphate reabsorption rate/glomerular filtration rate; ULN = upper limit of normal.

^a^ Normal range according to the central laboratory 2.4–4.3 mg/dL (the value at baseline was below the LLN in 12/13 patients [92.3%]).

^b^ Normal range according to the central laboratory 8.5–10.2 mg/dL (the value at baseline was below the LLN in 1/13 patients [7.7%]).

^c^ Normal range according to the central laboratory 20–60 pg/mL (the value at baseline was below the LLN in 7/13 patients [53.8%]).

^d^ Normal range according to Fukumoto et al.^(^ ^27^ ^)^ ≥20 ng/mL (the value at baseline was below the LLN in 3/13 patients [23.1%]).

^e^ Normal range according to the central laboratory 10–65 pg/mL (the value at baseline was above the ULN in 11/13 patients [84.6%]).

^f^ Normal range according to Fukumoto et al.^(^ ^27^ ^)^ ≥30 pg/mL (the value at baseline was above the ULN in 13/13 patients [100%]).

^g^ Normal ranges according to Chong et al.^(^ ^7^ ^)^ are age‐ and sex‐dependent (the value at baseline was below the LLN in 13/13 patients [100%]).

^h^ Normal range according to the central laboratory 115–359 U/L (the value at baseline was above the ULN in 6/13 patients [46.2%]).

The mean dose of burosumab increased throughout the study period and at week 112 was 1.05 (min 0.0, max 2.0) mg/kg.

### Primary endpoint

**Fig 2:**
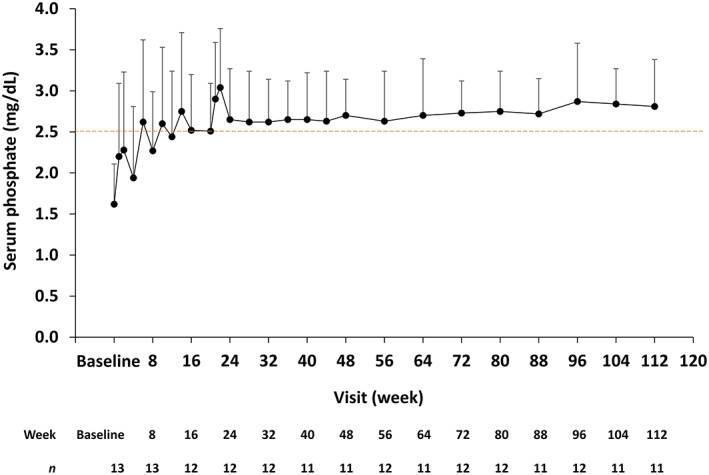
Serum phosphate level over time. Data are shown as mean + standard deviation. The lower limit of the normal range (2.5 mg/dL) is indicated. The number of observations at each time point are shown below the graph.

Serum phosphate levels increased after burosumab administration (Fig. 2). The mean ± SD serum phosphate level at baseline was 1.62 ± 0.49 mg/dL, which was below the LLN (2.5 mg/dL). After the first administration of burosumab at a dose of 0.3 mg/kg, mean serum phosphate levels increased and remained consistently above the LLN and in the normal range from week 14 to week 112.

### Secondary endpoints

Overall, 9/13 (69.2%) patients achieved mean peak serum phosphate levels above 2.5 mg/dL up to week 24. The mean change in peak levels from baseline was 1.01 ± 0.64 mg/dL (an increase of 62.9% from baseline). Similarly, 6/13 (46.2%) patients achieved mean trough serum phosphate levels above 2.5 mg/dL up to week 48. The mean change in trough levels was 0.88 ± 0.47 mg/dL (an increase of 58.2% from baseline). The proportion of patients with serum phosphate values within the normal range was 1/13 (7.7%) at baseline, 9/13 (69.2%) at week 24, 8/13 (61.5%) at week 48, and 9/13 (69.2%) at weeks 72 and 96.

Bone biopsy results were determined in 3 of the 4 patients who underwent the procedure, and the results are shown in Supplemental Table S1. Data from the other patient who underwent bone biopsy were not available because of the poor condition of the sample. No notable change was observed in osteoid thickness, osteoid/bone surface, and osteoid/bone volume (Supplemental Fig. S1). Mineralization lag time cannot be measured at baseline; mean ± SD value of mineralization lag time was 393.90 ± 487.62 at week 48.

**Fig 3:**
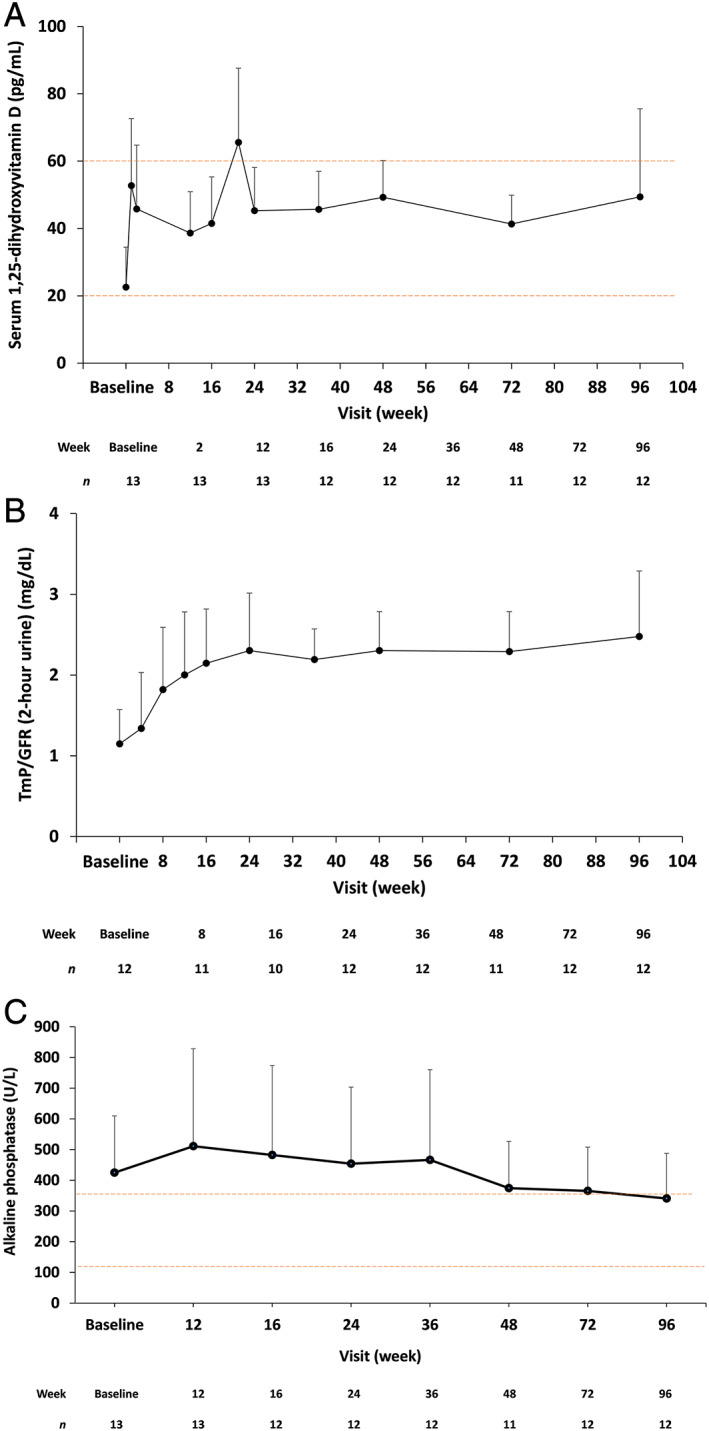
Pharmacodynamic parameters over time. (*A*) Serum 1,25‐dihydroxyvitamin D, (*B*) TmP/GFR, and (*C*) alkaline phosphatase. Data are shown as mean + standard deviation. For (*A*) serum 1,25‐dihydroxyvitamin D, normal range according to the central laboratory range of 20 to 60 pg/mL is indicated. For (*C*) alkaline phosphatase, normal range according to the central laboratory range of 115 to 359 U/L is indicated. The number of observations at each time point are shown below each graph. TmP/GFR = renal tubular maximum phosphate reabsorption rate/glomerular filtration rate.

The results of the pharmacodynamic marker analysis are summarized in Fig. 3. After the start of burosumab administration, mean ± SD serum 1,25(OH)_2_D levels increased from baseline (22.9 ± 11.9 pg/mL). Changes in serum 1,25(OH)_2_D levels at each time point were almost constant from week 24 onward. Similar results were obtained for TmP/GFR, which increased from baseline (1.15 ± 0.43 mg/dL) and remained almost constant from week 24 onward. Alkaline phosphatase levels increased from baseline (424.7 ± 184.8 U/L) to week 12 and then decreased below the baseline value from week 48 onward.

All metabolic bone markers initially increased after the start of burosumab administration (Supplemental Fig. S2). They reached maximum values at week 16 or 24 and then gradually decreased. The mean ± SD value of CTX was 0.61 ± 0.57 ng/mL at baseline; this increased to 0.89 ± 0.80 ng/mL at week 24, and then decreased to 0.78 ± 0.61 ng/mL at week 96. The mean ± SD values of P1NP at the same time points were 72.34 ± 72.87 ng/mL, 112.89 ± 105.43 ng/mL, and 91.77 ± 78.05 ng/mL, respectively. The mean ± SD values of BALP were 36.21 ± 21.66 μg/L, 39.17 ± 25.62 μg/L, and 26.52 ± 17.05 μg/L, respectively. The mean ± SD values of OC were 27.81 ± 28.85 ng/mL, 37.48 ± 37.88 ng/mL, and 35.18 ± 38.63 ng/mL, respectively.

The distance walked in the 6MWT increased after the start of burosumab administration (Table 2). The mean ± SD distance walked in the 6MWT was 295.8 ± 96.0 m at baseline, 329.2 ± 115.0 m (mean change: 33.0 m) at week 24, and 353.7 ± 115.8 m (mean change: 57.5 m, *p* < 0.05) at week 48. No notable changes were observed in the BPI throughout the study period (Table 2), with the exception of the worst pain score (question 3), which decreased: mean ± SD scores were 4.4 ± 2.6 at baseline, 3.0 ± 2.7 at week 24, 2.9 ± 2.9 at week 48, and 3.4 ± 2.5 at week 96.

**Table 2:** Assessment of Pain and Walking Distance Over Time

Assessment	Patients, *n*	Mean (SD)	Change from baseline, mean (SD)
Brief pain inventory—worst pain score			
Baseline	13	4.4 (2.6)	—
Week 24	12	3.0 (2.7)	−1.3 (1.7)
Week 48	12	2.9 (2.9)	−1.3 (2.8)
Week 96	12	3.4 (2.5)	−0.8 (1.5)
6‐minute walk test (m)			
Baseline	13	295.8 (96.0)	—
Week 12	13	314.2 (107.5)	18.4 (40.5)
Week 24	12	329.2 (115.0)	33.0 (44.8)
Week 48	12	353.7 (115.8)	57.5 (42.8)

SD = standard deviation.

### Exploratory endpoints

**Fig 4:**
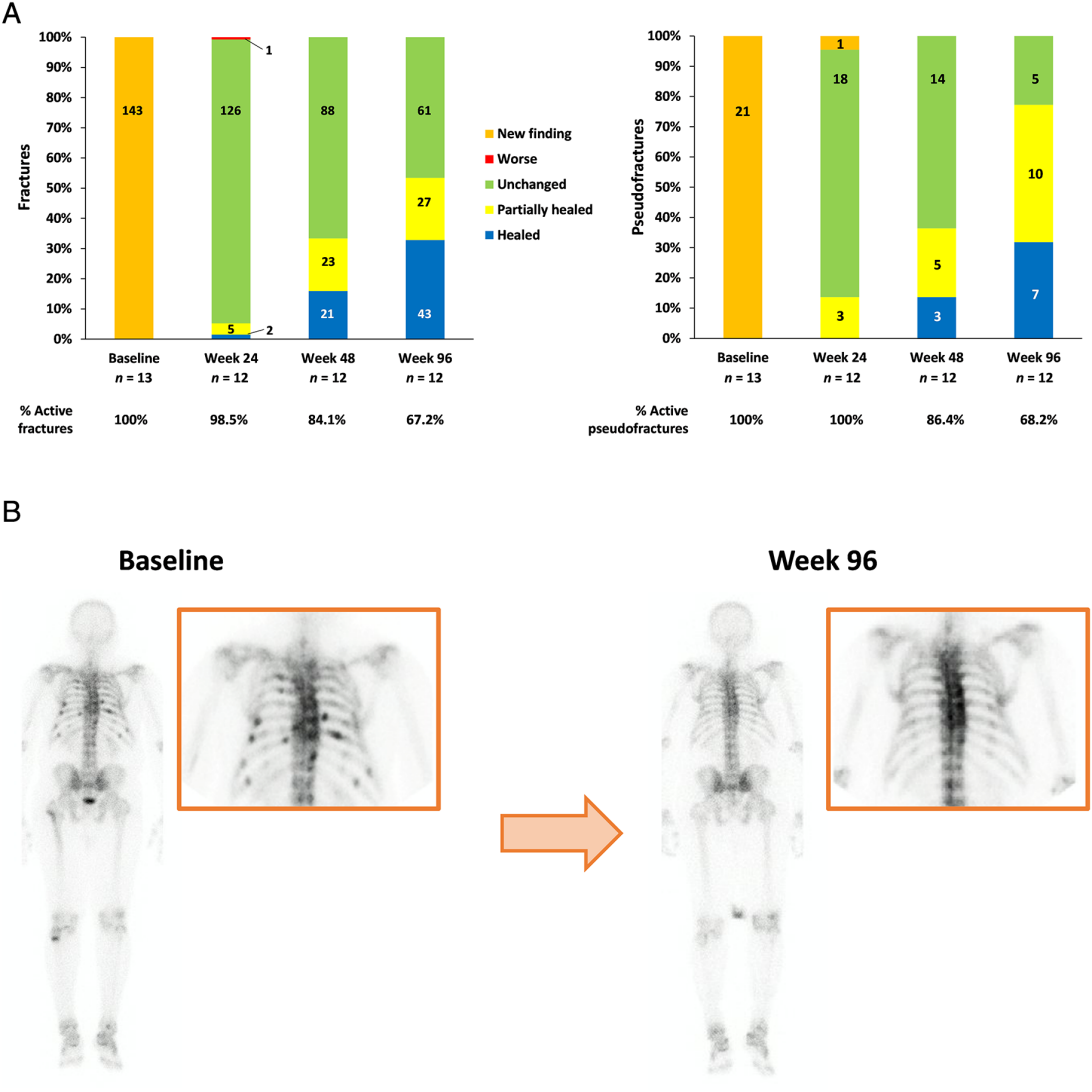
Outcomes of the ^99m^TC bone scan. (*A*) Evaluation of fractures and pseudofractures. (*B*) A ^99m^Tc‐labeled bone scan image of a 57‐year‐old male at baseline and at week 96. For the evaluation of fractures and pseudofractures, the % value (the percentage of active fractures remaining, ie, any status other than “healed”) was calculated as the count of each item/total count of each visit.

At baseline, a total of 143 active fractures and 21 active pseudofractures were observed by whole‐body bone scintigraphy. At week 48, the numbers of “healed” fractures and pseudofractures were 21/143 (14.7%) and 3/21 (14.3%), respectively, and the numbers of “partially healed” fractures and pseudofractures were 23/143 (16.1%) and 5/21 (23.8%), respectively (Fig. 4*A*). The numbers for “healed” at week 96 were 43/143 (30.1%) and 7/21 (33.3%) and those for “partially healed” were 27/143 (18.9%) and 10/21 (47.6%), respectively. Neither “worse” nor “new finding” fractures or pseudofractures were reported at week 48 or 96. An example ^99m^Tc‐labeled bone scan image is shown in Fig. 4*B*. The patient, a 57‐year‐old male, showed almost complete fracture healing at week 96.

DXA results over time are shown in Supplemental Fig. S3. The bone mineral density (BMD) of the lumbar spine was noticeably improved from baseline to week 96, as was the BMD of the total hip, although to a lesser extent.

### Safety

At the cut‐off date, 12/13 (92.3%) patients reported TEAEs. Most TEAEs were grade 1 or 2 in severity. Treatment‐related TEAEs were reported for 5 patients (38.5%); none were grade ≥3. There were no TEAEs leading to discontinuation or death. Four patients (30.8%) reported serious TEAEs (septic shock, infectious gastroenteritis, and large intestine polyp in 1 patient; large intestine polyp in 1 patient; herpes zoster in 1 patient; septic shock in 1 patient), but none were judged by the investigator to be related to study treatment. Table 3 shows the most frequently reported TEAEs (in ≥2 patients) and Supplemental Table S2 shows treatment‐related TEAEs.

**Table 3:** Summary of Treatment‐Emergent Adverse Events (Occurring in *n* ≥2 Patients)

TEAE, preferred term	Safety analysis set *N* = 13
Any TEAE	12 (92.3)
Events occurring in ≥2 patients	
Nasopharyngitis	8 (61.5)
Contusion	3 (23.1)
Eczema	3 (23.1)
Fatigue	3 (23.1)
Headache	3 (23.1)
Arthralgia	2 (15.4)
Cataract	2 (15.4)
Constipation	2 (15.4)
Cystitis	2 (15.4)
Dizziness	2 (15.4)
Herpes zoster	2 (15.4)
Large intestine polyp	2 (15.4)
Myalgia	2 (15.4)
Nausea	2 (15.4)
Rash	2 (15.4)
Septic shock	2 (15.4)
Tooth fracture	2 (15.4)

Data are shown as *n* (%). TEAE = treatment‐emergent adverse event.

Renal function data (serum creatinine and eGFR) are reported in Supplemental Fig. S4. No notable changes were observed during the study period, indicating that burosumab treatment was not associated with alterations of renal function. Values related to calcium homeostasis (serum corrected calcium, 24‐hour urine calcium, and intact parathyroid hormone) are shown in Supplemental Fig. S5. No changes in any calcium‐related parameters were observed. On renal ultrasound, nephrocalcinosis was reported (score >0) for 4 patients at baseline through to week 48, but no patient had an increase of >1 point. Nephrolithiasis was reported for 1 patient, with the first observation at week 24; it was no longer present at week 96.

Abnormal echocardiogram findings were reported for 2 patients. Anterior‐septal partial hypokinesis was reported at baseline and week 24 for 1 patient. For the second patient, who had chronic heart failure complications, left ventricular dysfunction, left atrial dilatation, and left ventricular mild mitral regurgitation were reported at baseline, and left ventricular asynergy and mild mitral regurgitation were reported at weeks 24 and 48.

With regard to immunogenicity, 1 patient tested positive for anti‐burosumab binding antibody at baseline, but at all other visits these were negative for all patients; no neutralizing antibodies were detected at any time. No other instances of anti‐burosumab binding antibody or neutralizing antibody were detected at any point. Tumor images were obtained from 7 patients by magnetic resonance imaging. No events of tumor progression were reported.

## Discussion

This clinical study, conducted in Japan and the Republic of Korea, was the first to examine the outcomes of burosumab treatment in TIO patients. Although most of the patients had been previously treated with active vitamin D and/or inorganic phosphate, the interim study results showed that serum phosphate levels increased after burosumab administration. In summary, 69.2% of patients achieved mean peak serum phosphate levels above the LLN and 46.2% of patients achieved mean trough levels above the LLN; the distance walked in the 6MWT increased after the start of burosumab administration; worst pain scores decreased; and the number of fractures and pseudofractures was reduced. These findings suggest the efficacy of burosumab in TIO patients who commonly have higher FGF23 concentrations^(^ ^24^ ^)^ and elevated bone biomarker levels^(^ ^25^ ^)^ compared with XLH patients.

The only previous clinical study in TIO patients with burosumab was the phase 2 study in the US.^(^ ^20^ ^)^ The results of the 2 studies were similar, confirming that burosumab was able to increase levels of serum phosphate, serum 1,25(OH)_2_D, and TmP/GFR. In both studies, bone biomarkers initially increased after burosumab initiation but gradually decreased after week 16. In our study, worst pain scores improved from baseline, and the distance walked in the 6MWT increased after burosumab treatment initiation. Taken together, these data suggest burosumab has the potential to restore normal phosphate levels and could provide clinical benefit to patients with TIO, a group for whom adequate treatment is currently lacking.

Of note, in the present study, the improvement in BPI worst pain score was not as marked as we would have expected considering the evidence of fracture healing. One of the possible explanations for this is that during treatment with burosumab, bone fractures and pseudofractures improved with an increase of serum phosphate concentration, and BPI pain tended to improve with time. However, the enrolled patients had an average age of 60.5 years and an average duration of disease of 10.0 years. Therefore, these patients were likely to have a wide range of complications other than TIO that may have contributed to their pain. Another possible reason is that the baseline BPI worst pain score in this study was relatively low at 4.4, whereas in a global study targeting XLH patients,^(^ ^26^ ^)^ the baseline BPI worst pain score was higher at 6.7. Therefore, the relatively low BPI worst pain scores at baseline in the patients enrolled in the present study may have led to minor changes with burosumab administration.

In the present study, the efficacy of burosumab by histomorphometry data could not be confirmed because the number of cases was small. However, when evaluating individual data, of the 3 patients with very severe osteomalacia, 1 patient showed no changes in histomorphometry parameters or serum phosphate levels by week 48. The other 2 patients showed an increase in serum phosphate levels and slight improvement in bone biopsy parameters.

Burosumab administration was initiated at a low dose (0.3 mg/kg) in this study, and titrated intra‐individually according to the serum phosphate level. The mean dose after week 52 was approximately 1.0 mg/kg. However, the optimal dose of burosumab in TIO varied widely, and approximately half of TIO patients required higher doses compared with XLH patients.^(^ ^15^, ^16^ ^)^

The treatment of choice for patients with TIO is surgical resection of the causative tumor.^(^ ^4^ ^)^ Surgical resection may still be more effective when compared with burosumab treatment, although conducting comparative analyses would be difficult because of the small patient population. However, in patients with unresectable tumors or those in whom causative tumors are not found, currently available treatments have had limited efficacy in healing the osteomalacia, reducing symptoms, and maintaining serum phosphate, parathyroid hormone, and alkaline phosphatase levels within the normal range;^(4^ ^)^ thus, burosumab may satisfy the unmet need for this population. All of the patients enrolled in this study had previously used conventional therapy (oral inorganic phosphate and/or active vitamin D_3_). From this, given that a proportion of fractures and pseudofractures were found to have healed or improved after the initiation of burosumab, we can speculate that burosumab may be more effective than current treatment, although it must be remembered that direct comparisons were not conducted in this study.

Burosumab was generally well tolerated, with no treatment‐related TEAEs of grade ≥3 and no severe AEs. Similarly, in the US study in patients with TIO, treatment‐related AEs were mild in severity.^(^ ^20^ ^)^ Two patients discontinued during our study, 1 because of disease progression and 1 who withdrew consent. No clinically relevant changes in serum calcium, urinary calcium, or intact parathyroid hormone were observed throughout this study, and no clinically significant evidence of hyperphosphatemia or ectopic renal calcification by ultrasound was observed.

The following study limitations must be considered when evaluating the data. First, this study was a single‐arm, open‐label study in Japanese and Korean TIO patients and did not include a comparative control group. Second, only 13 patients were enrolled in this study and received burosumab. However, the evaluation of fracture/pseudofracture and bone biopsy samples was performed by an independent central reviewer and was considered reliable, increasing the robustness of the study.

In this first report of burosumab to treat patients with TIO, these interim study results indicate that burosumab exhibits an acceptable safety profile and has the potential to provide clinical benefit for patients with unresectable tumors. The full results of the study will be the subject of a future article and are eagerly anticipated.

## Disclosures

YI has received research grants and consulting fees from Kyowa Kirin Co., Ltd. YTakeuchi and SF have received consulting fees from Kyowa Kirin Co., Ltd. NI has received research grants from Kyowa Kirin Co., Ltd. YR, CSS, YTakahashi, MM, and YS have no conflicts of interest to report. HO, MKojima, MKanematsu, and HK are employees of Kyowa Kirin Co., Ltd.

### Peer Review

The peer review history for this article is available at https://publons.com/publon/10.1002/jbmr.4184.

## Supporting information


**Appendix S1.** Supporting Information
