# Supplementary material for: Interim Analysis of a Phase 2 Open‐Label Trial Assessing Burosumab Efficacy and Safety in Patients With Tumor‐Induced Osteomalacia
Source: J Bone Miner Res. 2020 Nov 4;36(2):262–70. doi: 10.1002/jbmr.4184 (PMC7988547; doi:10.1002/jbmr.4184)
Supplement: Supplementary file 1 — Appendix S1. Supporting Information [file JBMR-36-262-s001.docx]

**Supplementary Data**

**Supplementary Table 1.** Summary of histomorphometric parameters

|  | Baseline | Week 48 | Difference | Percent change |
| --- | --- | --- | --- | --- |
| Osteoid thickness, μm |  |  |  |  |
| n | 3 | 3 | 3 | 3 |
| Mean (SD) | 16.03 (13.67) | 13.50 (7.05) | −2.53 (8.18) | 1.67 (48.33) |
| Median (range) | 8.70 (7.6–31.8) | 13.60 (6.4–20.5) | −1.20 (−11.3–4.9) | −15.80 (−35.5–56.3) |
| Osteoid/bone surface ratio, % |  |  |  |  |
| n | 3 | 3 | 3 | 3 |
| Mean (SD) | 47.7 (23.1) | 43.0 (5.6) | −4.7 (19.8) | 1.40 (38.66) |
| Median (range) | 38.0 (31–74) | 44.0 (37–48) | −1.0 (−26–13) | −2.60 (−35.1–41.9) |
| Osteoid/bone volume ratio, % |  |  |  |  |
| n | 3 | 3 | 3 | 3 |
| Mean (SD) | 14.03 (15.23) | 9.23 (5.48) | −4.80 (10.14) | −4.73 (41.33) |
| Median (range) | 7.10 (3.5–31.5) | 8.60 (4.1–15.0) | 0.60 (−16.5–1.5) | 17.10 (−52.4–21.1) |
| Mineralization lag time, days |  |  |  |  |
| n | - | 2 | - | - |
| Mean (SD) | - | 393.90 (487.62) | - | - |
| Median (range) | - | 393.90 (49.1–738.7) | - | - |

SD = standard deviation.

**Supplementary Table 2.** Summary of treatment-related treatment-emergent adverse events

| TEAE, preferred term | Safety analysis set  N = 13 |
| --- | --- |
| Any treatment-related TEAE | 5 (38.5) |
| Abdominal pain | 1 (7.7) |
| Arthralgia | 1 (7.7) |
| Cataract | 1 (7.7) |
| Dry eye | 1 (7.7) |
| Fatigue | 1 (7.7) |
| Glaucoma | 1 (7.7) |
| Hypoesthesia | 1 (7.7) |
| Injection site hypersensitivity | 1 (7.7) |
| Insomnia | 1 (7.7) |
| Myalgia | 1 (7.7) |
| Rash | 1 (7.7) |
| Toothache | 1 (7.7) |
| Vertigo | 1 (7.7) |

Data are shown as n (%).

TEAE = treatment-emergent adverse event.

**Supplementary Fig. 1.** Change from baseline in bone histomorphometric parameters for the three patients with evaluable bone biopsy data.

Orange lines indicate the normal range (based on Recker RR, Kimmel DB, Parfitt AM, et al. Static and tetracycline-based bone histomorphometric data from 34 normal postmenopausal females. J Bone Miner Res. 1988;3:133–44).

**Supplementary Fig. 2.** Percent change from baseline in serum bone biomarkers. (A) bone specific alkaline phosphatase (BALP), (B) procollagen 1 N-terminal propeptide (P1NP), (C) osteocalcin, (D) type I collagen C-telopeptides (CTX).

Data are shown as mean + standard deviation.

The number of observations at each time point are shown below each graph.

**Supplementary Fig. 3.** Percent change from baseline in lumbar spine and total hip DXA scores (BMD % change)

Data are shown as mean +/− standard deviation.

BMD = bone mineral density; DXA = dual-energy X-ray absorptiometry.

**Supplementary Fig. 4.** Renal parameters over time: (A) creatinine and (B) eGFR

Data are shown as mean + standard deviation.

The number of observations at each time point are shown below each graph.

eGFR = estimated glomerular filtration rate.

**Supplementary Fig. 5.** Calcium homeostasis over time. (A) Serum calcium, (B) 24-hour urinary calcium, and (C) intact parathyroid hormone (PTH)

The number of observations at each time point are shown below each graph.
